# Enterovirus 71 infection in children with hand, foot, and mouth disease in Shanghai, China: epidemiology, clinical feature and diagnosis

**DOI:** 10.1186/s12985-015-0308-2

**Published:** 2015-06-03

**Authors:** Ying Wang, Gang Zou, Aimei Xia, Xiangshi Wang, Jiehao Cai, Qianqian Gao, Shilin Yuan, Guimei He, Shuyi Zhang, Mei Zeng, Ralf Altmeyer

**Affiliations:** Institute of Molecular Ecology and Evolution, SKLEC & IECR, East China Normal University, Shanghai, 200062 China; Department of Infectious Diseases, Children’s Hospital of Fudan University, Shanghai, 201102 China; Unit of Anti-infective Research, Key Laboratory of Molecular Virology & Immunology, Institut Pasteur of Shanghai, Chinese Academy of Sciences, Shanghai, 200031 China

**Keywords:** Enterovirus 71, Hand, foot and mouth disease, VP1 sequence, Neutralizing antibody, EV71 diagnosis

## Abstract

**Background:**

In 2012 a large outbreak of hand, foot, and mouth disease (HFMD) widely spread over China, causing more than 2 million cases and 567 deaths. Our purpose was to characterize the major pathogens responsible for the 2012 HFMD outbreak and analyze the genetic characterization of the enterovirus 71 (EV71) strains in Shanghai; also, to analyze the dynamic patterns of neutralizing antibody (NAb) against EV71 and evaluate the diagnostic value of several methods for clinical detection of EV71.

**Methods:**

Clinical samples including stool, serum and CSF were collected from 396 enrolled HFMD inpatients during the peak seasons in 2012. We analyzed the molecular epidemiology, clinical feature, and diagnostic tests of EV71 infection.

**Results:**

EV71 was responsible for 60.35 % of HFMD inpatients and 88.46 % of severe cases. The circulating EV71 strains belonged to subgenogroup C4a. The nucleotide sequences of VP1 between severe cases and uncomplicated cases shared 99.2 ~ 100 % of homology. Among 218 cases with EV71 infection, 211 (96.79 %) serum samples showed NAb positive against EV71 and NAb titer reached higher level 3 days after disease onset. Of 92 cases with EV71-associated meningitis or encephalitis, 5 (5.43 %) of 92 had EV71 RNA detected in CSF samples. The blood anti-EV71 IgM assay showed a sensitivity of 93.30 % and a specificity of 50 %.

**Conclusions:**

EV71 C4a remained the predominant subgenotype circulating in Shanghai. The severity of the EV71 infection is not associated with the virulence determinants in VP1. RT-PCR together with IgM detection can enhance the early diagnosis of severe EV71-associated HFMD.

**Electronic supplementary material:**

The online version of this article (doi:10.1186/s12985-015-0308-2) contains supplementary material, which is available to authorized users.

## Background

Hand, foot, and mouth disease (HFMD) has been an emerging highly contagious disease in children in countries of the Western Pacific Region since the late nineties, posing a serious public health threat. HFMD is usually a benign self-limited illness among young children characterized by fever, ulcerating vesicles in the mouth and macula or papulovesicular on hands, feet and buttocks [1–4]. However, a small proportion of the affected children may develop neurological and cardiopulmonary complications with high case fatality rates [5, 6]. The mainland China has been experiencing the persistent national outbreaks of HFMD since 2008. According to the data from the national surveillance system of HFMD in mainland China in 2008–2012, the case-fatality rate of HFMD is 0.03 % and the case-severity rate is 1.1 % [7]. HFMD is caused by enteroviruses belonging to the species *Enterovirus A* (serotypes Coxsackie virus A 2–8, 10, 12, 14, 16 and enterovirus 71, 76 and 89–92), and rarely by *Enterovirus B,* with CA16 and EV71 as the main causative agents [8, 9]. In China, EV71 infection has been associated with large outbreaks of HFMD and the majority of fatalities.

EV71, first isolated in 1969, is a single, positively-stranded RNA virus without envelope [10, 11]. The VP1 protein of EV71 contains a number of important neutralization epitopes and has been extensively used for molecular typing [12–14]. EV71 can be phylogenetically classified into 3 main genogroups (A, B and C) and 11 genotypes (A, B1 ~ B5 and C1 ~ C5) based on VP1 sequences [15, 16]. Monitoring the genetic variations of circulating EV71 strains and the emergence of new types or recombinants of EV71 in the epidemic regions is important for vaccine development and drug discovery.

In 2012, 2,198,442 HFMD cases and 567 deaths-associated with HFMD were reported in mainland China, with the number of the notifiable cases and the fatal cases being 33.90 % and 11.39 % higher than those in 2011, respectively [7]. Meanwhile, the outbreaks of EV71 infection and HFMD were also reported in Malaysia, Thailand, and Cambodia in 2012 [17–19]. In this study, we collected feces, serum and CSF specimens from 396 HFMD inpatients in Shanghai during the 2012 peak season. Our purpose was to characterize the major pathogens responsible for this outbreak and to analyze the genetic characterization of the EV71 strains. Besides, we analyzed the dynamic patterns of neutralizing antibody (NAb) titer against EV71 and evaluated the diagnostic value of several methods for the detection of EV71 in clinical samples.

## Results

### Clinical description

A total of 396 inpatients with HFMD including 292 (73.7 %) uncomplicated cases and 104 (26.3 %) severe cases were enrolled into this study from March 28th to July 5th in 2012, representing 47.2 % of all hospitalized HFMD cases (778) at the CHFU during the study period. The 104 severe cases included 94 (92.16 %) aseptic meningitis, 4 (3.85 %) encephalitis, 3 (2.94 %) encephalomyelitis and 3 (2.94 %) brain stem encephalitis with neurogenic pulmonary hemorrhage/edema. Three severe cases with laboratory-confirmed EV71 infections were fatal with 2 female infants (both were 7 months) succumbing to cardiopulmonary failure and 1 female child (5.7 years) succumbing to central respiratory failure.

Of the 396 patients, the male-to-female ratio was 1.75:1 and the age range was between 3 months and 12.7 years with the median age of 29 months old and 357 (90.15 %) cases <5 years old, of whom, 1- to 2-year-old children accounted for 127 (32.07 %) cases. Persistent fever >3 days, vomiting, startle, myoclonus and higher positive rate of EV71-RNA in stool were significantly more frequent in severe cases (Table 1).

**Table 1:** Comparison of clinical manifestations between uncomplicated cases and severe cases

	All cases	Uncomplicated	Severe	*Χ* ^2^	P value*
	(396)	(73.74 %, 292)	(26.26 %,104)		
Male to female ratio	1.75 (252/144)	1.92 (192/100)	1.36 (60/44)	2.153	0.155
Fever > 3 days	16.41 % (65)	11.64 % (34)	29.81 % (31)	18.441	**<0.0001**
Oral rash	88.38 % (350)	89.38 %(261)	85.58 % (89)	1.082	0.291
Vomiting	45.45 % (180)	38.70 % (113)	64.42 % (67)	20.468	**<0.0001**
Headache	2.53 % (10)	2.05 % (6)	3.85 % (4)	1.000	0.298
Startle	57.32 % (227)	46.92 % (137)	86.54 % (90)	49.209	**<0.0001**
Convulsion	1.26 % (5)	0.68 % (2)	2.88 %(3)	2.976	0.116
Myoclonus	35.10 % (139)	21.92 % (64)	72.12 % (75)	84.826	**<0.0001**
EV71-RNA positive in stool	242 (61.11 %)	143 (48.97 %)	99 (95.19 %)	68.933	**<0.0001**

*Chi-squared test was used to evaluate the significant variation of clinical manifestations between uncomplicated cases and severe cases. The p value less than 0.05 was considered to be significant and marked in bold

### EV71 detection in the stool samples by real-time RT-PCR

EV71 was identified in 239 (60.35 %) cases, CA16 was identified in 82 (20.71 %) cases, EV71 and CA16 were co-detected in 3 (0.758 %) uncomplicated cases and the remaining 72 (18.18 %) cases contained untyped enteroviruses. Among the 104 severe cases, 92 (88.46 %) were positive for EV71 while only 149 (51.02 %) positive for EV71 in 292 uncomplicated cases, revealing that EV71 was more frequently in severe cases than in uncomplicated cases (*χ*^2^ = 45.113, p < 0.0001).

### EV71 detection in blood samples

**Fig. 1:**
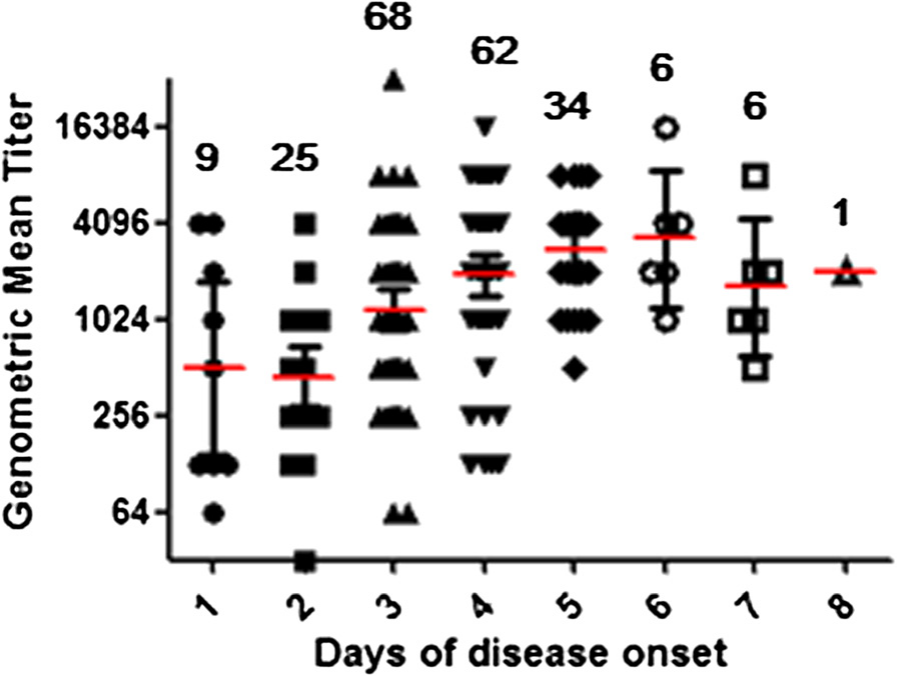
Neutralizing antibody titers in HFMD inpatients after days of disease onset

The average blood sampling days were 3.4 ± 1.3 days (range: 1–8 days) after disease onset. Among 218 cases with EV71 positivity in stool samples, 211 (96.79 %) were positive for NAb against EV71; meanwhile, among 138 cases with EV71 negativity in stool samples, 31 (22.46 %) were also positive for NAb against EV71. We analyzed the levels of NAb titers in sera from patients with EV71-RNA positivity in their stool samples. As shown in Fig. 1, we found that NAb GMT significantly increased with the time of disease onset (*χ*2 = 46.10, p < 0.0001) and reached the highest level at day 6. Although NAb GMT decreased at day 7 and day 8 after disease onset, there was no significant difference in NAb GMT at day 6, day 7 and day 8 after disease onset (*χ*2 = 1.912, p = 0.385).

Of 200 serum samples obtained from the patients with EV71 RNA positive in stool samples and positive NAb against EV71, 151 (75.5 %) were positive for EV71 RNA in sera. Serum EV71 RNA was detected positive in 44 (60.27 %) of 73 severe cases and 107 (84.25 %) of 127 uncomplicated cases, respectively (*χ*2 = 14.41, p = 0.0001). The NAb GMT was 1122.5 and 1622.6 in 73 severe cases and 127 uncomplicated cases, respectively (*χ*2 = 4.084, p = 0.043), indicating there was no difference in the NAb titer level between severe cases and uncomplicated cases. The average serum sampling days after disease onset was 3.18 ± 1.04 days (range: 1–5 days) in severe cases and 3.9 ± 1.34 days in uncomplicated cases (range: 1–8 days) (*χ*2 = 15.90, p < 0.0001), respectively.

Of the enrolled 396 patients, 267 who had both EV71-RNA detected in stool samples and NAb detected in serum samples had EV71 IgM tested in whole blood samples from finger tip. Compared to the EV71 infection confirmed by both positive EV71-RNA in stool and positive serum NAb against EV71, blood EV71 IgM showed a sensitivity of 93.30 % and a specificity of 50 %; the positive predict value was 79.15 % and the negative predict value was 78.57 % (Table 2).

**Table 2:** Evaluation of blood EV71 IgM rapid test kit

Blood EV71-IgM antibody	stool EV71-RNA and serum NAb against EV71		
	Both positive	Both negative	Total
Positive	167	44	211
Negative	12	44	56
Total	179	88	267

### Phylogenetic analyses of VP1 gene of EV71

The entire VP1 region of EV71 was amplified using RT-PCR directly from the clinical serum and CSF specimens rather than from cultured isolates to avoid adaptive mutations selected during serial passage. EV71 RNA was detected in 5 (5.43 %) of 92 CSF samples obtained from the severe patients with EV71 RNA positive in the stool samples. The PCR products of 151 EV71-positive serum samples and 5 EV71-positive CSF samples could be sequenced successfully. The EV71 VP1 nucleotide and amino acid sequence among the 2012 Shanghai strains showed 99.2 ~ 99.8 % and 98.9 ~ 99.6 % identity, respectively. Based on the VP1 sequences, the nucleotide and amino acid homologies between severe cases and uncomplicated cases were 99.2 ~ 100 % and 98.9 ~ 100 %, respectively.

**Fig. 2:**
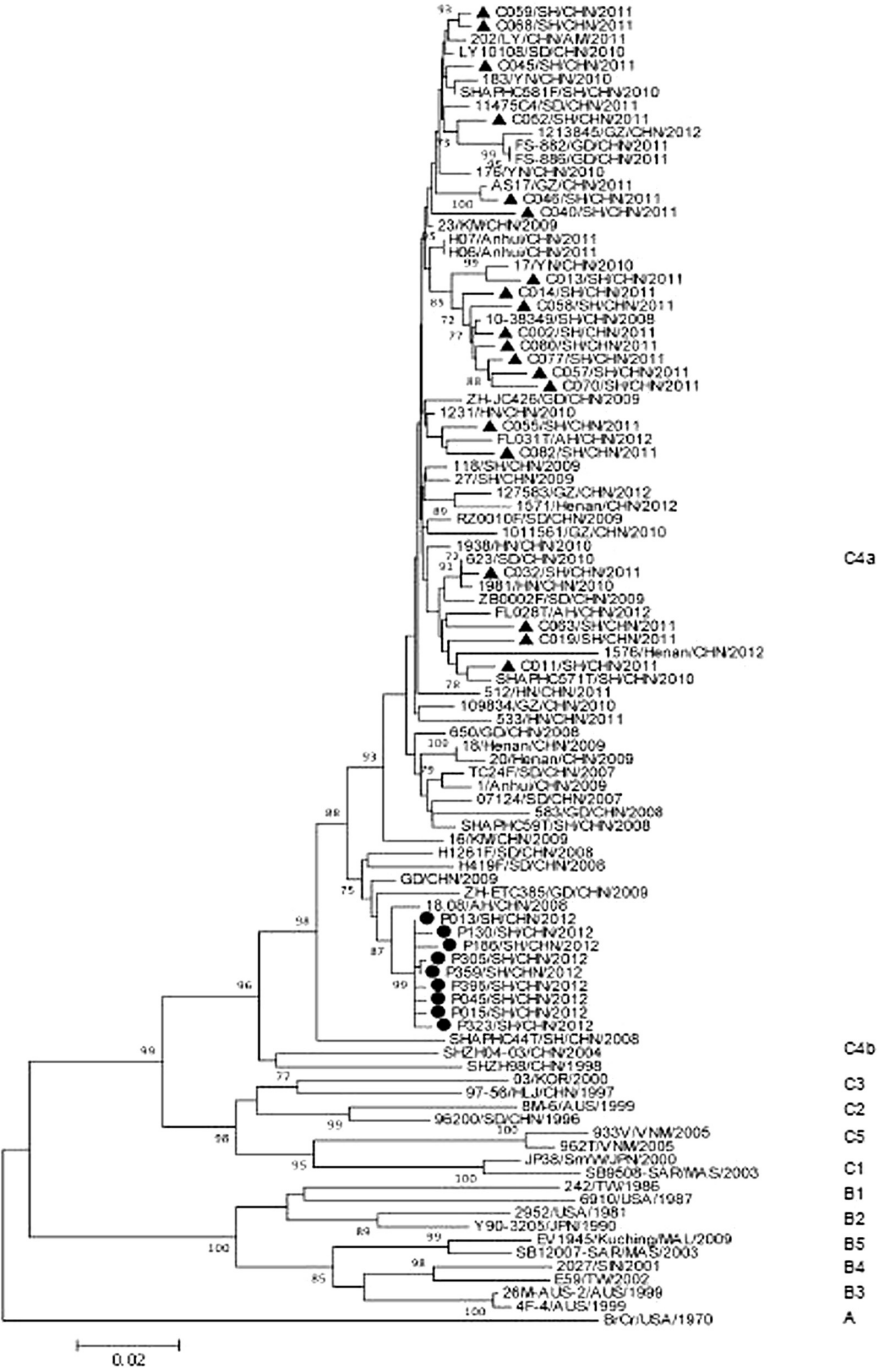
Phylogenetic analysis of EV71 from Shanghai, 2012. The phylogenetic tree was based on the alignment of entire VP1 sequences using Neighbor-Joining method with bootstrap value 1000 pseudoreplicates. Bootstrap values with > 75 replications were shown at the branch nodes. The scale bar represented the genetic distance of 0.02 nucleotide substitutions per site. ▲ indicated EV71 strains from Shanghai, 2011, ● indicated EV71 strains from Shanghai, 2012. Other strains are obtained from GenBank (listed in Additional file 1)

A phylogenetic tree was constructed based on VP1 sequences to determine the subgroups and subgenotypes of EV71 strains. Seventy reference sequences were obtained from GenBank, including 11 subgenotypes (A, B1-B5, and C1-C5) and representative strains epidemic in China since 2007. In addition, another twenty-six VP1 sequences of EV71 from 2008 to 2011 isolated in Shanghai were also used for alignment analyses. As shown in Fig. 2, all the Shanghai sequences were classified into the C4a cluster which was predominant since the 2008 HFMD outbreak in mainland China. The 2012 Shanghai EV71 strains shared a high degree of nucleotide homology (98.5 ~ 99.8 %) with 2008 Anhui strain 18.08/AH/CHN/2008. It should be noted that the nine 2012 Shanghai strains formed a separated subcluster (99 % bootstrap value) of the C4a clade from the 2011 Shanghai strains fell within the same cluster as the EV71 strains isolated from Guangdong, Shandong and Anhui regions in China during 2008–2009, sharing 96.63 ~ 98.88 % nucleotide and 98.32 ~ 99.66 % amino acid homologies, respectively. The VP1 sequences of 2012 EV71 strains circulating in Shanghai shared 93.94-99.33 % nucleotide and 96.97-98.99 % amino acid homologies with the 2008–2011 strains.

## Discussion

Although multiple genotypes and subgenotype of EV71 are circulating simultaneously or alternatively in other countries and regions, [20, 21] our study revealed that EV71 remained the predominant enterovirus causing the 2012 large outbreaks of HFMD and severe central nerves system (CNS) disease in Shanghai. Combined with our previous surveillance data, [22, 23] we reason that the principal strategy of controlling the HFMD outbreak in Shanghai is to prevent the EV71 circulation in children through mass EV71 vaccination. In line with previous studies, we also found that persistent fever >3 days, vomiting, startle, and myoclonus can be used as the warning signs for predicting the severe HFMD cases [8, 24].

We noted that the VP1 sequences of EV71 strains from severe cases were indistinguishable from those causing uncomplicated symptoms, which is same as other reports [11, 25, 26]. Besides, the VP1 sequences of EV71 strains in the serum and CSF specimens collected from the same patients were identical, suggesting the individual patient appeared to be infected with a single genotype of EV71. Phylogenetic analysis suggests that the constant genetic evolution of the EV71 strains circulating in Shanghai occurred in the VP1 gene over the year. It deserves to further explore the effect of the genetic evolution of VP1 on the antigen change of EV71 [27, 28]. Previous studies have reported that the residue L97 and domain 215-KQEKD-219 on VP1 protein of EV71 were associated with neutralizing epitope. We found that these neutralizing epitopes were unchanged in all the Shanghai strains, making the development and long-term use of EV71 vaccine feasible [29, 30].

Neutralization antibody plays an important role in immunoprotection from viral infection as it can efficiently bind virus, neutralize virus, and thus prevent the further progression of disease. It is usually believed that NAb produces and reaches the peak level during the late convalescent stage of viral infection. Based on these theories, IVIG containing NAb against EV71 is recommended for treating the severe EV71-associated diseases in many countries including our countries [21, 31]. Clinical observational study from Malaysia showed early IVIG therapy in severe EV71-associated HFMD patients might have a beneficial effect on halting disease progression and improve the outcomes [32]. Also, a mice model study showed that plasma containing high titer EV71-sepcific IVIG products can confer protection against lethal EV71 challenge [33]. However, we observed that serum NAb against EV71 has produced in over 95 % of EV71-infected HFMD patients when the HFMD-related symptoms appear and reaches the high level within 3–6 days after disease onset. In clinical setting, severe cases were admitted to hospital usually on day 3 after disease onset when NAb has already reached the high level. Therefore, our data suggest that if IVIG has any benefit in the treatment of HFMD, it is likely not due to the direct antivrial effect of EV71 neutralizing Abs. An immunomodulatory effect of IVIG has been proposed by In vitro data [34].

We detected 5 EV71 strains in 92 CSF samples using RT-PCR method and obtained the sequences of EV71. However, EV71 could not be isolated from CSF samples after 5 passages in RD cells. Obviously, CSF samples are not suitable to be used as the prior clinical samples for the diagnosis of EV71-associated CNS infections. Other studies reported the difficulty in isolating EV71 from CSF due to low amount of viral load, sensitivity of PCR, and intrathecal antibody in CSF [5, 22, 35, 36]. Real-time RT-PCR and viral isolation are considered as the standard methods in clinical diagnosis of viral infection. Although real-time RT-PCR has the advantage of high sensitivity and specificity in the diagnosis of EV71 infection in clinical samples, it takes 2–3 h to get the result and requires the expensive equipment in the hospital laboratory. Also, virus isolation is time-consuming and insensitive. The fatal HFMD was almost always caused by EV71 infection and the severe disease progress is very fast, thus, timely and rapid diagnosis of EV71 infection for suspected severe HFMD is critical for clinical management and early intervention [37]. Thus, a rapid, sensitive and convenient bedside diagnostic tool is needed in clinical practice. Blood EV71-IgM-Colloidal Gold immunochromatographic assay is easy to perform and provides the result within 15 min. It has high sensitivity and negative predict value but low specificity and positive predict value, therefore, EV71-IgM-Colloidal Gold assay as the confirmatory test for diagnosing EV71 infection was not recommended. However, given the high sensitivity and convenience, finger tip blood EV71 IgM test can be used as a rapid point-of-care screen tool and an essential supplement for the early diagnosis of EV71 infection to help clinicians timely recognize severe EV71-associated diseases.

The upcoming EV71 vaccination in China is a promising strategy to prevent the large outbreak of HFMD. Early recognition of progressive symptoms and timely diagnosis of EV71 infection in suspected severe HFMD will help take appropriate clinical intervention to reduce the fatality through intensive care. Ongoing surveillance of HFMD and EV71 infections and evolution of EV71 strains is necessary for control of HFMD in China and the formulation of EV71 vaccine before and after vaccine available in the market.

## Conclusions

This study revealed that EV71 remained the predominant enterovirus causing the 2012 large outbreaks of HFMD and severe CNS disease in Shanghai. We observed the constant genetic evolution of EV7 VP1 over the year. In fact, serum NAb against EV71 in HFMD patients has reached higher level 3 days after disease onset. Real-time RT-PCR together with IgM detection would enhance the detection of HFMD and thereby should be highly recommended in clinical therapy. Our findings provide important epidemiological information of HFMD epidemic in Shanghai and evaluate the issues regarding the laboratory diagnosis of EV71 infection.

## Methods

### Specimen and data collection

Clinical specimens used in this study were collected from hospitalized HFMD patients during the 2012 peak season (March 28th – July 5th) of HFMD in Shanghai at the Children’s Hospital of Fudan University (CHFU). The study was approved by the Ethics Committee of the study hospital. Both stool samples and serum samples were collected within 24 h after admission and a CSF sample was collected if a patient was highly suspected to have central nervous involvement and had a successful lumbar puncture procedure for the routine CSF diagnostic tests. A severe case was defined by HFMD accompanied with at least one of the following complications: aseptic meningitis, encephalitis, acute flaccid paralysis, pulmonary edema or hemorrhage, cardiopulmonary collapse. CSF pleocytosis was defined as a white blood cell count more than 10 × 10^6^ cells/L in a patient older than 1 month of age. A total of 263 serum specimens were collected from 292 uncomplicated HFMD patients, and 93 serum and 92 CSF specimens were collected from 104 severe HFMD patients. All the samples were stored at −80 °C for further tests. Clinical data were recorded using case-report form (CRF) and re-assessed by an infectious disease specialist.

### Detection of EV71 infection in clinical laboratory

EV71 detection in stool specimens was performed using commercially available pan-enterovirus, EV71, and CA16 diagnostic kit (Da An Gene Co., Ltd, Guangzhou, China) according to manufacturer’s instructions. Anti-EV71 IgM was detected in the peripheral finger tip blood sample using EV71-IgM-Colloidal Gold immunochromatographic assay (Beijing Wantai Biological Pharmacy Enterprise Co., Ltd., China).

### RT-PCR detection, virus isolation, and neutralization assay

Viral RNA was extracted from serum and CSF specimens using a QIAamp Viral RNA Mini Kit (Qiagen, Hilden, Germany). Direct RT-PCR was performed using SuperScript III One-Step System with Platinum Taq High Fidelity (Invitrogen, USA) with primer targeting EV71 VP1 gene (2372 F: 5’-GCAGCCCAAAAGAACTTCAC-3’, and 3454R: 5’-AAGTCGCGAGAGCTGTCTTC-3’) [1]. The resulting 1083-bp PCR product was analyzed on a 1 % agarose gel, and then purified using TIANgel Midi Purification Kit (TIANGEN, China) for sequencing. Of 92 CSF samples, 5 were positive using direct RT-PCR. However, virus isolation for 92 CSF samples using the strategy described in previous study was unsuccessful [38]. Neutralization assay was performed using the method described in previous study [39].

### Sequencing of EV71 strains and phylogenetic analyses

The gel purified PCR products were bi-directionally sequenced using an ABI 3730xl automatic DNA analyzer. Alignment of the entire VP1 nucleotide sequences (891 bp) of the EV71 strain was performed using Clustal W program. Phylogenetic tree was constructed in MEGA 5.2 (Molecular Evolutionary Genetics Analysis software; Tamura, Dudley, Nei, and Kumar 2012) using Neighbor-joining method in Kimura two-parameter model, accompanied by bootstrap analyses with 1000 replicates. The entire VP1 nucleotide sequences of the nine Shanghai EV71 strains and reference sequences obtained from GenBank were listed with accession numbers in Additional file 1.

### Statistical analyses

The data were analyzed using Microsoft Excel 2010, SPSS software version 19.0 and GraphPad Prism V5.01 software. The percentage of clinical data was analyzed using the chi-square test. NAb titers were log-transformed to geometric mean titers (GMT) for statistical analysis. GMT and the mean sampling days were analyzed using Kruskal Wallis test. The p value less than 0.05 was considered to be statistically significant.

## Additional file

Additional file 1:
**The list of EV71 strains used for phylogenetic analysis in this study.**
