# Supplementary material for: Enterovirus 71 infection in children with hand, foot, and mouth disease in Shanghai, China: epidemiology, clinical feature and diagnosis
Source: Virol J. 2015 Jun 3;12:83. doi: 10.1186/s12985-015-0308-2 (PMC4464242; doi:10.1186/s12985-015-0308-2)
Supplement: Additional file 1: — The list of EV71 strains used for phylogenetic analysis in this study. [file 12985_2015_308_MOESM1_ESM.pdf]

Additional file 1

Table S1. The list of EV71 strains used for phylogenetic analysis in this study.

Strain	Source	GenBank no.	Genotype
**P013/SH/CHN/2012**	This study	KJ139450	C4a
**P015/SH/CHN/2012**	This study	KJ159896	C4a
**P045/SH/CHN/2012**	This study	KJ159894	C4a
**P130/SH/CHN/2012**	This study	KJ159895	C4a
**P186/SH/CHN/2012**	This study	KJ159897	C4a
**P305/SH/CHN/2012**	This study	KJ159898	C4a
**P323/SH/CHN/2012**	This study	KJ159900	C4a
**P359/SH/CHN/2012**	This study	KJ159899	C4a
**P395/SH/CHN/2012**	This study	KJ159901	C4a
**C002/SH/CHN/2011**	This study	KJ188634	C4a
**C011/SH/CHN/2011**	This study	KJ188629	C4a
**C013/SH/CHN/2011**	This study	KJ188628	C4a
**C014/SH/CHN/2011**	This study	KJ188627	C4a
**C019/SH/CHN/2011**	This study	KJ188630	C4a
**C032/SH/CHN/2011**	This study	KJ188631	C4a
**C040/SH/CHN/2011**	This study	KJ188635	C4a
**C045/SH/CHN/2011**	This study	KJ188636	C4a
**C046/SH/CHN/2011**	This study	KJ188633	C4a
**C052/SH/CHN/2011**	This study	KJ188626	C4a
**C055/SH/CHN/2011**	This study	KJ188625	C4a
**C057/SH/CHN/2011**	This study	KJ188624	C4a
**C058/SH/CHN/2011**	This study	KJ188623	C4a
**C059/SH/CHN/2011**	This study	KJ188632	C4a
**C063/SH/CHN/2011**	This study	KJ188640	C4a
**C068/SH/CHN/2011**	This study	KJ188639	C4a

**C070/SH/CHN/2011**	This study	KJ188638	C4a
**C077SH/CHN/2011**	This study	KJ188637	C4a
**C080/SH/CHN/2011**	This study	KJ188642	C4a
**C082/SH/CHN/2011**	This study	KJ188641	C4a
**242/TW/1986**	GenBank	JN874548	B1
**18_08/AH/CHN/2008**	GenBank	GQ121427	C4a
**07124/SD/CHN/2007**	GenBank	JQ326299	C4a
**GD/CHN/2009**	GenBank	JF799986	C4a
**10-38349/SH/CHN/2008**	GenBank	JQ766173	C4a
**TC24F/SD/CHN/2007**	GenBank	EU753418	C4a
**17/YN/CHN/2010**	GenBank	JN251929	C4a
**SHAPHC571T/SH/CHN/2010**	GenBank	JF918576	C4a
**623/SD/CHN/2010**	GenBank	HQ668404	C4a
**SHAPHC581F/SH/CHN/2010**	GenBank	JF918578	C4a
**650/GD/CHN/2008**	GenBank	HM037808	C4a
**AS17/GZ/CHN/2011**	GenBank	JX203260	C4a
**583/GD/CHN/2008**	GenBank	HM037805	C4a
**LY10108/SD/CHN/2009**	GenBank	KF853532	C4a
**H1261F/SD/CHN/2008**	GenBank	GQ253399	C4a
**H419F/SD/CHN/2008**	GenBank	GQ253394	C4a
**SHAPHC59T/SH/CHN/2008**	GenBank	HM579943	C4a
**SHAPHC44T/SH/CHN/2008**	GenBank	HM579942	C4a
**1231/HN/CHN/2011**	GenBank	HQ668453	C4a
**BrCr/USA/1970**	GenBank	JN874547	A
**1/Anhui/CHN/2009**	GenBank	GQ994988	C4a
**ZH-ETC385/GD/CHN/2009**	GenBank	GQ487686	C4a
**ZH-JC426/GD/CHN/2009**	GenBank	GQ487689	C4a
**18/Henan/CHN/2009**	GenBank	KM288747	C4a
**20/Henan/CHN/2009**	GenBank	KM288749	C4a

**ZB0002F/SD/CHN/2009**	GenBank	GQ253419	C4a
**RZ0010F/SD/CHN/2009**	GenBank	GQ253417	C4a
**118/SH/CHN/2009**	GenBank	HQ667787	C4a
**27/SH/CHN/2009**	GenBank	HQ667783	C4a
**23/KM/CHN/2009**	GenBank	JF505392	C4a
**16/KM/CHN/2009**	GenBank	JF505391	C4a
**Y90-3205/JPN/1990**	GenBank	AB433863	B2
**1011561/GZ/CHN/2010**	GenBank	KJ865523	C4a
**109834/GZ/CHN/2010**	GenBank	KJ865517	C4a
**1938/HN/CHN/2010**	GenBank	KM260010	C4a
**1981/HN/CHN/2010**	GenBank	KM260011	C4a
**26M-AUS-2/AUS/1999**	GenBank	AF376101	B3
**183/YN/CHN/2010**	GenBank	AB725679	C4a
**176/YN/CHN/2010**	GenBank	AB725678	C4a
**H07/Anhui/CHN/2011**	GenBank	HQ328793	C4a
**H06/Anhui/CHN/2011**	GenBank	HQ328792	C4a
**E59/TW/2002**	GenBank	JN874551	B4
**FS-882/GD/CHN/2011**	GenBank	KC801034	C4a
**FS-886/GD/CHN/2011**	GenBank	KC801033	C4a
**533/HN/CHN/2011**	GenBank	KM260020	C4a
**512/HN/CHN/2011**	GenBank	KM260019	C4a
**202/LY/CHN/AM/2011**	GenBank	KF150174	C4a
**11475C4/SD/CHN/2011**	GenBank	KJ772432	C4a
**EV1945/Kuching/MAL/2009**	GenBank	HM358835	B5
**FL031T/AH/CHN/2012**	GenBank	KF925272	C4a
**FL028T/AH/CHN/2012**	GenBank	KF925271	C4a
**1213845/GZ/CHN/2012**	GenBank	KJ865525	C4a
**127583/GZ/CHN/2012**	GenBank	KJ865520	C4a
**1576/Henan/CHN/2012**	GenBank	KM260024	C4a

**1571/Henan/CHN/2012**	GenBank	KM260022	C4a
**96200/SD/CHN/1996**	GenBank	JQ326306	C2
**97-56/HLJ/CHN/1997**	GenBank	AB115494	C3
**JP38/Sm/W/JPN/2000**	GenBank	HQ676217	C1
**SB12007-SAR/MAS/2003**	GenBank	AY905548	B5
**4F-4/AUS/1999**	GenBank	AF376105	B3
**2027/SIN/2001**	GenBank	JQ766153	B4
**8M-6/AUS/1999**	GenBank	AF376109	C2
**2952/USA/1981**	GenBank	AF135888	B2
**933V/VNM/2005**	GenBank	AM490161	C5
**03/KOR/2000**	GenBank	DQ341356	C3
**6910/USA/1987**	GenBank	AF135901	B1
**962T/VNM/2005**	GenBank	AM490162	C5
**SB9508-SAR/MAS/2003**	GenBank	AY258301	C1
**SHZH98/CHN/1998**	GenBank	AF302996	C4b
**SHZH04-3/CHN/2004**	GenBank	AY895142	C4b
